# Ecchymosis in the Chest After Bilateral Sagittal Split Osteotomy and Genioplasty: A Case Report and Literature Review

**DOI:** 10.7759/cureus.104064

**Published:** 2026-02-22

**Authors:** AlJoharah AlShathry, Ohoud M Alotaibi, Shahad H Aati, Rodina F Aljamaan, Osama A Alharbi, Bader Fatani, Mohammed A Salah

**Affiliations:** 1 Dentistry, King Saud University, Riyadh, SAU; 2 Oral and Maxillofacial Surgery, Prince Sultan Military Medical City, Riyadh, SAU; 3 Oral and Maxillofacial Surgery, Riyadh Second Health Cluster, Riyadh, SAU; 4 Oral and Maxillofacial Surgery, King Saud University, Riyadh, SAU; 5 Oral and Maxillofacial Surgery, University Dental Hospital, Riyadh, SAU

**Keywords:** bilateral sagittal split osteotomy, complication, ecchymosis, jaw surgery, mandible

## Abstract

Bilateral sagittal split osteotomy (BSSO) is a corrective jaw surgery that repositions the mandible. Potential complications include unexpected fractures, unfavorable splits, avascular necrosis, condylar resorption, and malposition of the proximal segment. Chest ecchymosis following BSSO is a rare complication. Ecchymosis, the leakage of blood into subcutaneous tissues, typically occurs near the surgical site, such as the face or neck. This can result from arterial damage during surgery or postoperative factors, such as the formation of a hematoma. In this case report, we demonstrate a case of an unusual post-surgical complication of chest ecchymosis following BSSO and genioplasty. The patient was further treated with analgesics, monitoring vitals and airway and encouraging oral intake and bed rest.

## Introduction

The development of chest ecchymosis after bilateral sagittal split osteotomy (BSSO) is a relatively rare complication, but it bears severe clinical consequences. BSSO is a common method of orthognathic surgery used to correct mandibular prognathism, retrognathism, and mandibular deformities and enhance facial aesthetics and function. Although BSSO is a relatively safe procedure, there are many risks for intraoperative and postoperative complications. The reported complications are nerve injury, poor skin sutures, and prolonged bleeding [1,2]. Extracranial complications, such as ecchymosis, especially in the chest, are rarely described in the literature. Ecchymosis, characterized by the extravasation of blood into subcutaneous tissues, typically manifests in areas near the surgical site, such as the face or neck [3]. This case report presents a patient who developed significant chest ecchymosis post BSSO and genioplasty. This may arise either due to damage of an artery at the time of surgery or postoperatively from iatrogenic factors, such as the formation of a hematoma. However, chest ecchymosis due to BSSO is very rare, as most injuries are localized at the jaw, face, or neck levels. Kantar et al. and Robl et al. reported a whole series of adverse events following the orthognathic intervention, extracranial ecchymosis being particularly unrecognized [1,2]. Verweij et al. highlighted various risk factors, such as patient age and intraoperative technique, that contribute to complications, but discussions on chest manifestations remain sparse [4]. Due to the peculiar nature of this complication, in the current case report, we will discuss its potential etiopathogenesis and treatment options, as well as their broader implications on clinical practice. We will provide a comprehensive review of the literature in an effort to improve understanding and shed light on how this rare presentation should be managed in future surgical practices. The aim of this case report is to describe a rare presentation of chest ecchymosis following BSSO and genioplasty, to highlight its possible etiopathogenesis and management, and to emphasize the importance of recognizing extracranial manifestations as part of postoperative monitoring after orthognathic surgery.

## Case presentation

**Figure 1:**
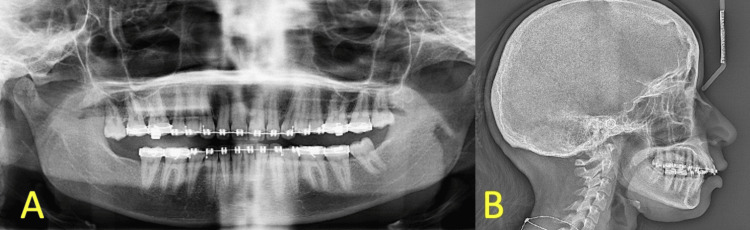
(A) Preop panoramic radiograph. (B) Preop lateral cephalometric radiograph.

A 25-year-old female patient, unaware of any medical history and with no known allergies, presented to the oral and maxillofacial surgery clinics with a class II skeletal relationship due to a retruded mandible. The patient came to the clinic seeking orthognathic surgery after she completed her orthodontic treatment. Preoperative panoramic radiograph and lateral cephalometric are demonstrated in Figure 1. 

The case was planned for BSSO with 9 mm advancement, genioplasty of 6 mm, and extraction of tooth #38. The patient was brought to the theater awake and oriented and placed on the table in a supine position, monitors were connected, anesthesia was introduced, and nasal intubation was performed on the right nostril. Eyes were lubricated and taped shut. Then, the patient was prepped and draped in a sterile manner. Lidocaine 2% with epinephrine was injected in all surgical sites. In the mandible, a mucosal incision is made over the ascending ramus. Subperiosteal dissection was done. Lingula and the inferior border were exposed. A BSSO cut was done medially over the ridge, and a buccal vertical cut down to the inferior border. Splitting was done, and the nerve was visualized and protected. The segments were fixed based on the final splint. Fixation using the KLS 2.0 plate with 7 mm screws. In the chin, an incision was made 5 mm below the mucogingival junction. Subperiosteal dissection was done. The mental nerve is exposed and protected bilaterally. Midline was marked, and splitting was done. The chin was advanced by 6 mm and fixed with a pre-bent plate using screws measuring 7-9 mm in length. The flap was repositioned and sutured in layers of muscle and mucosa with Vicryl 4-0.

**Figure 2:**
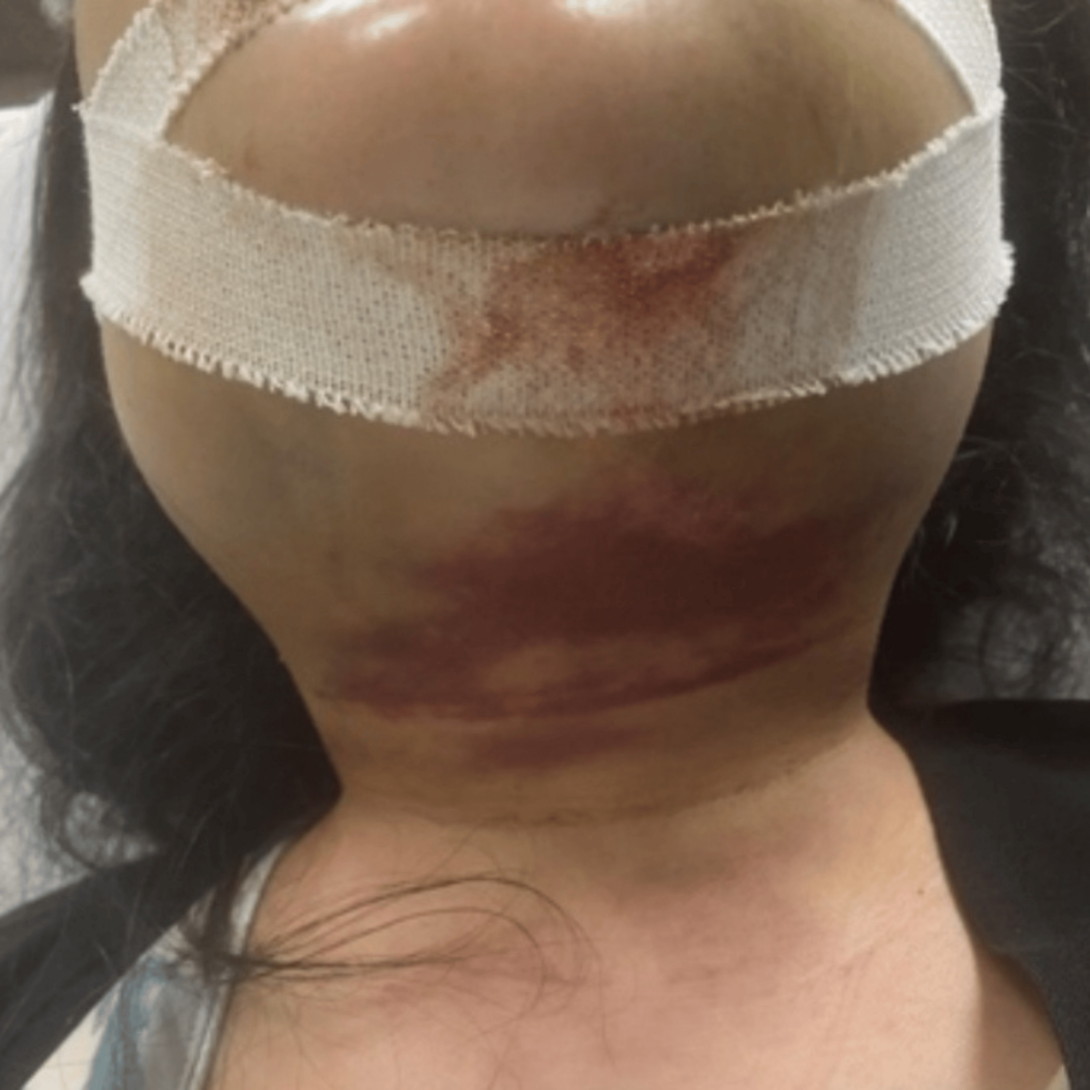
Submandibular ecchymosis that extended to the neck mid-way.

Three days post surgery, the patient presented with severe swelling in the mandible, a fever that reached 38 °C associated with sputum, blood with pus, and throat congestion, and an inability to tolerate her medications orally. Physical examination revealed bilateral soft and non-tender buccal edema and bilateral hard non-tender submandibular edema, along with ecchymosis that extended to the neck mid-way, which is shown in Figure 2.

**Figure 3:**
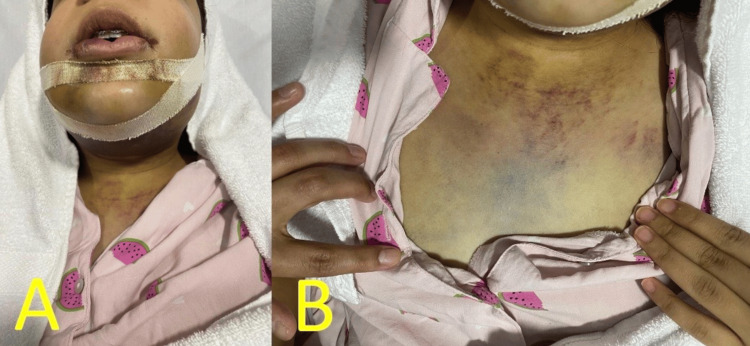
(A) Bilateral lower face edema. (B) Ecchymosis of the chest.

The intraoral examination could not be obtained due to limited mouth opening. The case was managed by monitoring of the airway, intravascular fluid, and antibiotics (Augmentin 1.2 gm q12hr). One day postoperatively, the facial swelling was still the same size; however, it was a softer consistency in the submandibular area, along with an extension of neck ecchymosis to the chest area, as shown in Figure 3.

Due to the unexpected extracranial extension of ecchymosis, a hematology consultation was obtained to exclude underlying bleeding or coagulation disorders. Laboratory investigations included complete blood count, prothrombin time, international normalized ratio, activated partial thromboplastin time, and inflammatory markers. All results were within normal limits.

Although postoperative ecchymosis is often self-limiting, the extent and progression observed in this patient warranted close clinical monitoring. Management remained conservative and included frequent vital sign monitoring, airway observation, adequate analgesia, encouragement of oral intake, and monitoring of fluid input and output. The patient was discharged one week following the admission. The ecchymosis gradually resolved with conservative management. The area of ecchymosis began to resolve within two weeks, with complete resolution by one month. The patient was followed weekly in the outpatient clinic, noting the gradual resolution of the ecchymosis. From the patient's perspective, the unexpected chest ecchymosis caused initial anxiety; however, reassurance and supportive management led to progressive resolution with satisfaction regarding the overall surgical outcome.

**Figure 4:**
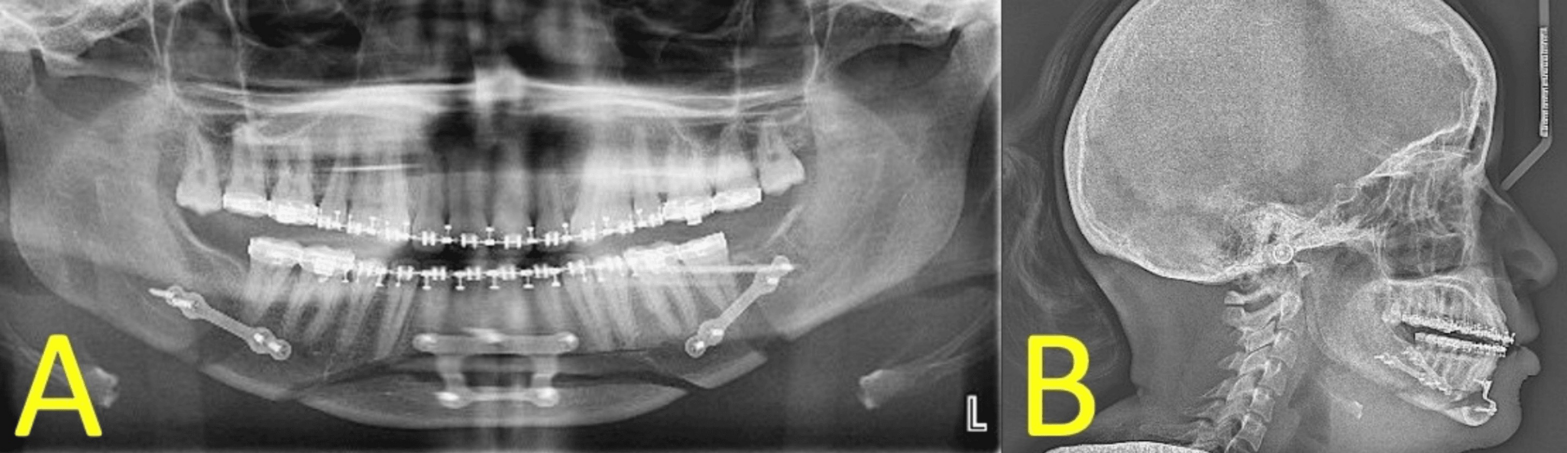
(A) Postop panoramic radiograph. (B) Postop lateral cephalometric radiograph.

Postop radiographic imaging is presented in Figure 4.

## Discussion

BSSO is associated with several well-documented complications, including neurosensory disturbances, infection, hemorrhage, unfavorable splits, and relapse [1,2]. Ecchymosis is a recognized postoperative finding; however, it is typically confined to the facial, submandibular, or cervical regions [1-3].

Chest ecchymosis following BSSO represents an extremely rare postoperative manifestation, with very limited reporting in the maxillofacial surgery literature. Most large clinical series and reviews of orthognathic surgery complications do not emphasize ecchymosis extending beyond the neck, rendering such extracranial presentations underrecognized [1,2,4].

A review of the literature demonstrates that complication rates following BSSO increase in more complex procedures, particularly bimaxillary surgeries and cases involving extensive soft tissue dissection or concomitant genioplasty [1,5-7]. Factors such as surgical technique, presence of third molars, and patient-related variables have been shown to influence postoperative complication rates [4-7].

The underlying mechanism of chest ecchymosis following mandibular osteotomy remains unclear. Proposed explanations include gravity-assisted tracking of blood through cervical fascial planes, vascular injury during osteotomy or fixation, and postoperative venous congestion [7,8]. These mechanisms remain hypothetical due to the rarity of reported cases, which limits definitive conclusions regarding the etiopathogenesis of this complication.

The findings add to the existing body of knowledge by identifying ecchymosis in the chest as a rare yet notable postoperative complication. Previous research largely focused on facial areas, but recent studies suggest that blood pooling in non-facial regions can occur due to vascular trauma during mandibular surgery [8-10]. This expands the understanding of the scope of postoperative issues and suggests that complications may not always be limited to the facial area.

The findings align with earlier research showing that complications such as infection, nerve damage, and bad splits are common. However, recent studies provide fresh perspectives on rarer complications such as chest ecchymosis, which older studies did not thoroughly address. This suggests a broader spectrum of potential complications than previously recognized [2,11,12].

The occurrence of chest ecchymosis challenges the traditional focus on facial complications and suggests that BSSO can have more widespread effects on the body, particularly affecting the vascular system [13]. Panula et al. reported similar unexpected postoperative findings in large clinical series [8]. This observation warrants a rethinking of postoperative monitoring strategies, particularly in more complex bimaxillary surgeries [14].

The development of chest ecchymosis was an unexpected finding and may be due to anatomical differences or unanticipated vascular injury during surgery. Alternative explanations could include the use of excessive pressure during surgery or preexisting clotting disorders that went undiagnosed. These factors require further investigation to determine why this complication arises [7,15].

The reviewed studies have several strengths, particularly those with large sample sizes, which offer a broad view of complication rates. However, limitations exist, such as potential biases in patient selection and the retrospective nature of many studies. Confounding factors, including the presence of third molars, also complicate the assessment of specific risks [2,13,16]. Additionally, the rarity of chest ecchymosis suggests that it may occur due to chance in some cases [17].

The recognition of rare complications such as chest ecchymosis highlights the need for enhanced intraoperative monitoring and postoperative care. The findings suggest that surgeons should consider the broader vascular impact of BSSO, not just the localized facial effects. Future research should aim to quantify the incidence of these rare complications and further explore their underlying physiological mechanisms [16,17].

## Conclusions

This case report highlights chest ecchymosis as a rare and unexpected complication following BSSO and genioplasty. Although BSSO is generally safe, this finding suggests that postoperative effects may extend beyond the facial region, likely involving vascular factors. Increased awareness of such uncommon complications is important for comprehensive postoperative monitoring and patient management. Further research is needed to clarify the underlying mechanisms and incidence of chest ecchymosis in orthognathic surgery. This case underscores the need for heightened awareness of rare extracranial complications following BSSO and genioplasty and supports comprehensive postoperative monitoring when unexpected clinical findings arise.
